# Intensity-modulated radiation therapy for definitive treatment of cervical cancer: a meta-analysis

**DOI:** 10.1186/s13014-018-1126-7

**Published:** 2018-09-14

**Authors:** Yanzhu Lin, Kai Chen, Zhiyuan Lu, Lei Zhao, Yalan Tao, Yi Ouyang, Xinping Cao

**Affiliations:** 1 Department of Radiation Oncology, Sun Yat-sen University Cancer Center, State Key Laboratory of Oncology in South China, Collaborative Innovation Center for Cancer Medicine, 651 Dongfeng Road East, Guangzhou, Guangdong 510060 People’s Republic of China; 2 Department of Oral and Maxillofacial Surgery, First Affiliated Hospital, Sun Yat-sen University, Guangzhou, 510080 People’s Republic of China

**Keywords:** Cervical cancer, IMRT, 3DCRT, 2DRT

## Abstract

**Background:**

To compare the efficacies and toxicities of intensity-modulated radiotherapy (IMRT) with three-dimensional conformal radiotherapy (3D-CRT) or conventional two-dimensional radiotherapy (2D-RT) for definitive treatment of cervical cancer.

**Methods:**

A meta-analysis was performed using search engines, including PubMed, Cochrane Library, Web of Science, and Elsevier. In the meta-analysis, odds ratios (ORs) were compared for overall survival (OS), disease-free survival (DFS), and acute and chronic toxicities.

**Results:**

Included data were analysed using RevMan 5.2 software. Six studies encompassing a total of 1008 patients who received definitive treatment (IMRT = 350, 3-DCRT/2D-RT = 658) were included in the analysis. A comparison of 3-year OS and 3-year DFS revealed no significant differences between IMRT and 3D-CRT or 2D-RT (3-year OS: OR = 2.41, 95% confidence interval [CI]: 0.62–9.39, *p* = 0.21; 3-year DFS: OR = 1.44, 95% CI: 0.69–3.01, *p* = 0.33). The incidence of acute gastrointestinal (GI) toxicity and genitourinary (GU) toxicity in patients who received IMRT was significantly lower than that in the control group (GI: Grade 2: OR = 0.5, 95% CI: 0.28–0.89, *p* = 0.02; Grade 3 or higher: OR = 0.55, 95% CI: 0.32–0.95, *p* = 0.03; GU: Grade 2: OR = 0.41, 95% CI: 0.2–0.84; *p* = 0.01; Grade 3 or higher: OR = 0.31, 95% CI: 0.14–0.67, *p* = 0.003). Moreover, the IMRT patients experienced fewer incidences of chronic GU toxicity than did the control group (Grade 3: OR = 0.09, 95% CI: 0.01–0.67, p = 0.02).

**Conclusion:**

IMRT and conventional radiotherapy demonstrated equivalent efficacy in terms of 3-year OS and DFS. Additionally, IMRT significantly reduced acute GI and GU toxicities as well as chronic GU toxicity in patients with cervical cancer.

## Background

Cervical cancer is the second most common malignant tumour in women and is the third leading cause of cancer-related death among women worldwide [1]. Thus, it represents a serious threat to women’s health. The incidence and mortality of cervical cancer in China are the highest in the world. Radical surgery and radiotherapy (RT) are equally efficacious in the treatment of patients with stage I–IIA cervical cancer [2].

External beam radiation combined with intracavitary brachytherapy is the main RT approach for locally advanced cervical carcinoma. In the past few decades, conventional two-dimensional RT (2D-RT) has been widely used in the treatment of cervical cancer, but this treatment option suffers from a high frequency of acute and chronic complications, which affect the treatment efficacy as well as patient quality of life [3]. Three-dimensional conformal RT (3D-CRT) based on computed tomography is becoming a critical part of RT. This approach is relatively favourable in terms of the radiation dose and toxicity to organs in the exposure field [4].

Intensity-modulated RT (IMRT) is a precise RT that has been developed on the basis of 3D-CRT [5]. An advantage of IMRT is that it can deliver a relatively large radiation dose over a target area while minimising the radiation dose to adjacent noncancerous tissue, thereby offering greater locoregional control and leading to fewer side effects. IMRT is associated with lower gastrointestinal and haematological toxicities than is conventional RT (c-RT) in treatment of cervical cancer, and it is therefore used more widely [6, 7]. However, the potential advantages of IMRT for treating cervical cancer remain unclear. Therefore, this meta-analysis evaluated whether IMRT results in more favourable clinical outcomes than 2D-RT or 3D-CRT do in patients with intact cervical cancer in terms of overall survival (OS) and toxicity.

## Methods

### Search strategy

This analysis strictly followed the guidelines of the Preferred Reporting Items for Systematic Reviews and Meta-Analyses (PRISMA) statement [8]. The analysis was performed on studies with publication dates up to 13 February 2018. Several search engines (PubMed, the Cochrane Library, Web of Science, and Elsevier) were used to identify articles that investigated the relationship between IMRT and conventional RT or 3D conformal RT for treating cervical cancer. The keywords used were as follows: [intensity-modulated OR conformal OR dimensional OR 2D OR 3D] AND [radiotherapy* OR radiation therapy] AND [cervical OR cervix OR uterine] AND [tumour OR cancer OR carcinoma]. Only English-language publications were included.

### Inclusion and exclusion criteria

Studies were selected for inclusion in this analysis according to the following selection criteria: 1) Study participants were patients with cervical cancer who were diagnosed by pathological examination. 2) IMRT was compared with 3D-CRT or 2D-RT in previously untreated patients, and the efficacy was reported. 3) Patients were treated with RT and concurrent chemotherapy. 4) The number of participants in the experimental group was ≥10. Exclusion criteria were as follows: 1) Case reports, conference abstracts, comments, and letters to the editors were excluded. 2) Studies based on patients who had received previous surgical treatment for cervical cancer were excluded. 3) Duplicate publications were excluded.

### Data extraction

Two reviewers extracted data from each eligible study. Information extracted from eligible studies included the first author’s name, year of publication, study design, and number of study participants, as well as participant age, region, cancer stage, RT dose, and major outcomes. Disagreements were resolved through discussion and consensus.

### Data analysis and statistical methods

All statistical analyses were performed using RevMan 5.2 (Cochrane Collaboration, Oxford, UK). All survival outcomes and toxicity measurements from the studies were analysed based on odd ratios (ORs) with a 95% confidence interval (CI). The heterogeneity among studies was assessed using chi square or *I*^2^ statistics (*p* < 0.1 indicated significant heterogeneity). If *I*^2^ > 50% or *p* < 0.1, the results for the chi-squared tests were considered statistically significant, and a random-effects model was chosen. Otherwise, we used a fixed-effects model (the Mantel–Haenszel method) for further evaluations. The pooled effect size was significant if *p* < 0.05.

## Results

### Literature search

**Fig. 1:**
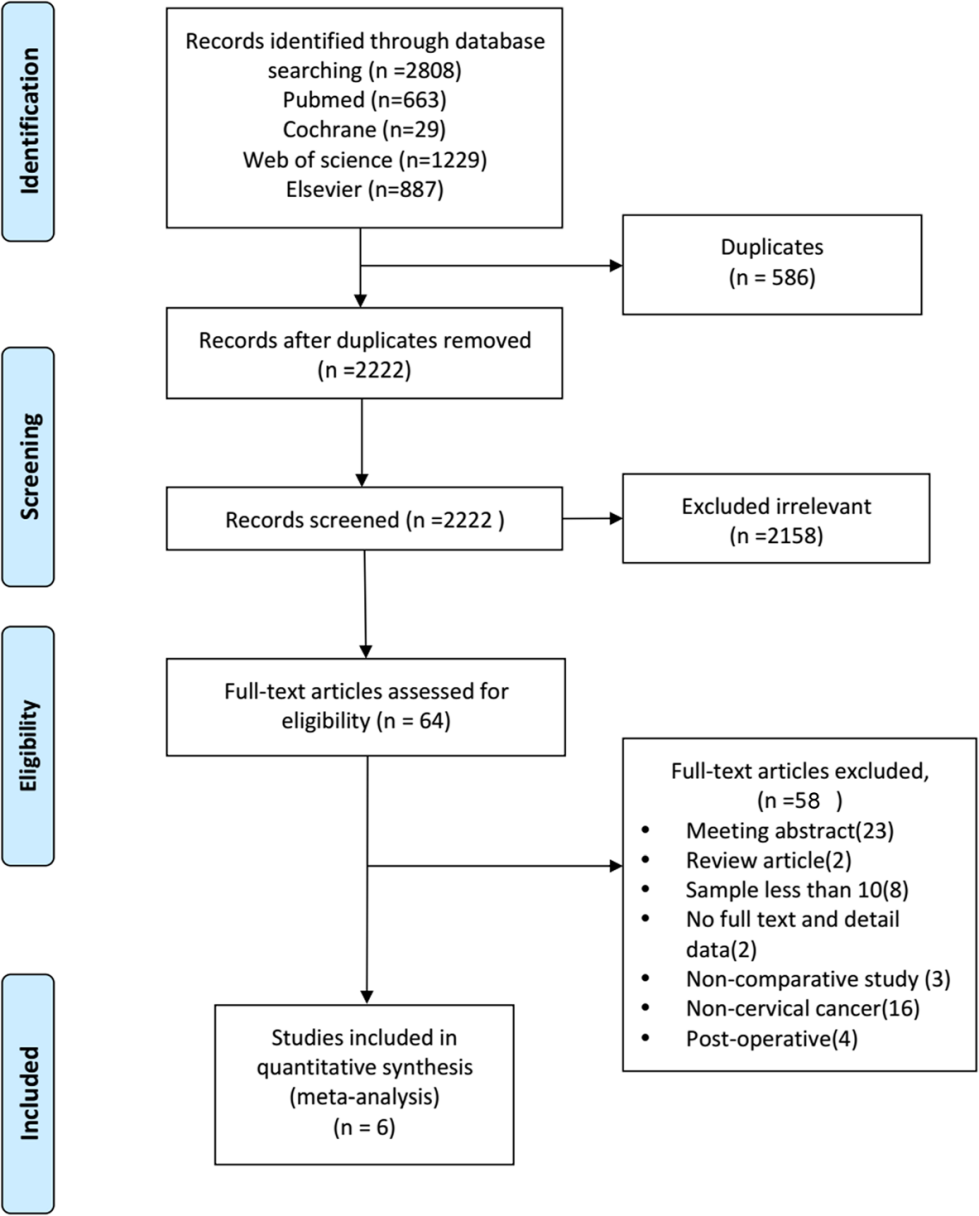
PRISMA flow diagram of study selection

The initial literature search based on the keywords yielded 2808 articles. After examination for and exclusion of duplicate and irrelevant articles, 64 articles remained for full-text review. The full texts of the potentially eligible articles were read, and six publications [9–14] were included in the meta-analysis. The detailed article selection process and exclusion criteria are presented in Fig. 1.

### Basic characteristics of the included studies

In total, six articles, encompassing 1008 participants (350 IMRT, 658 CRT), comparing the RT effects of IMRT and 3D-CRT or 2DRT were included in the meta-analysis. The detailed characteristics of the six eligible studies are presented in Table 1. Included studies were published in 2010 or thereafter. Geographically, five trials were conducted in Eastern countries, and one was conducted in the United States. The patients were aged 24–88 years. All the patients were treated with whole pelvis RT in combination with brachytherapy. The range of doses for external beam irradiation was 45–50 Gy. All treated patients were also receiving cisplatin-based chemotherapy at the time of RT treatment.

**Table 1:** Characteristics of all the included studies

Author	Year	Country	Study design	Stage	Treatment	Patients (n)	Median age (range)	RT doses (Gy)	Chemotherapy
Naik et al.	2016	India	Prospective	IIA-IIIB	IMRT	20	48(28–70)	50	cisplatin
					3D	20	45(30–75)		
Wu et al.	2016	Taiwan.	Retrospective	IB1-IVB	IMRT	30	80.5 (75–88)	45–50.4	cisplatin
					2D/3D	30	77.8 (75–88)		
Ganhdi et al.	2013	India	Prospective	IIB-IIIB	IMRT	22	50(36–65)	50.4	cisplatin
					2D	22	45(36–65)		
Chen et al.	2013	Taiwan	Retrospective	IB2-IIIB	IMRT	83	54	45	cisplatin
					2D/3D	237	54		
Du et al.	2012	China	Retrospective	IIB-IIIB	IMRT	60	52(31–74)	45–50	cisplatin
					2D	62	55(26–77)		
Kidd et al.	2010	USA	Prospective	IA2-IVB	IMRT	135	52	50	cisplatin
					2D/3D	317	52		

*n* number of patients, *RT* Radiotherapy, *2D* Two-dimensional, *3D* Three-dimensional, *IMRT* Intensity modulated RT

### Clinical outcomes

**Fig. 2:**
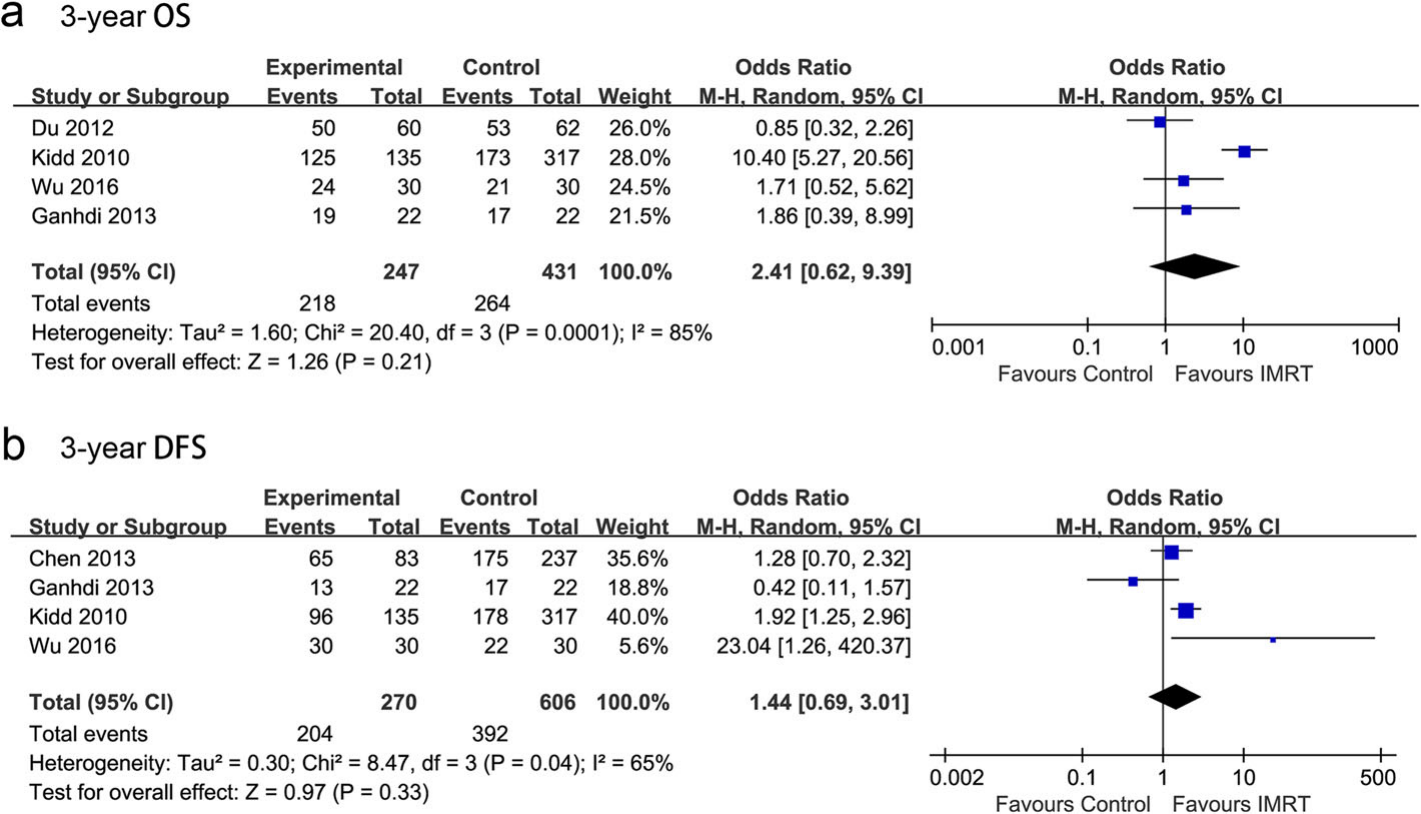
Comparison between IMRT and 2D-RT/3D-CRT for OS and DFS

Four of the included trials, accounting for 678 participants, reported 3-year OS data (Fig. 2a). Heterogeneity existed between the studies, and thus a random-effects model was chosen. The pooled OR for 3-year OS was 2.41, and the 95% CI was 0.62 to 9.39 (*p* = 0.21). The results suggested that patients with cervical cancer in the IMRT group and the 3D-CRT or 2D-RT groups did not exhibit significant differences with respect to 3-year OS.

Data regarding disease-free survival (DFS) were available in four studies. Heterogeneity existed between two of the studies (Chi^2^ = 8.47, *I*^*2*^ = 65%); therefore, a random-effects model was chosen. The pooled estimate of the OR was 1.44, and the 95% CI was 0.69 to 3.01 (*p* = 0.33). A random-effect meta-analysis indicated no difference between the two groups in terms of 3-year DFS (Fig. 2b).

### Acute toxicity

**Fig. 3:**
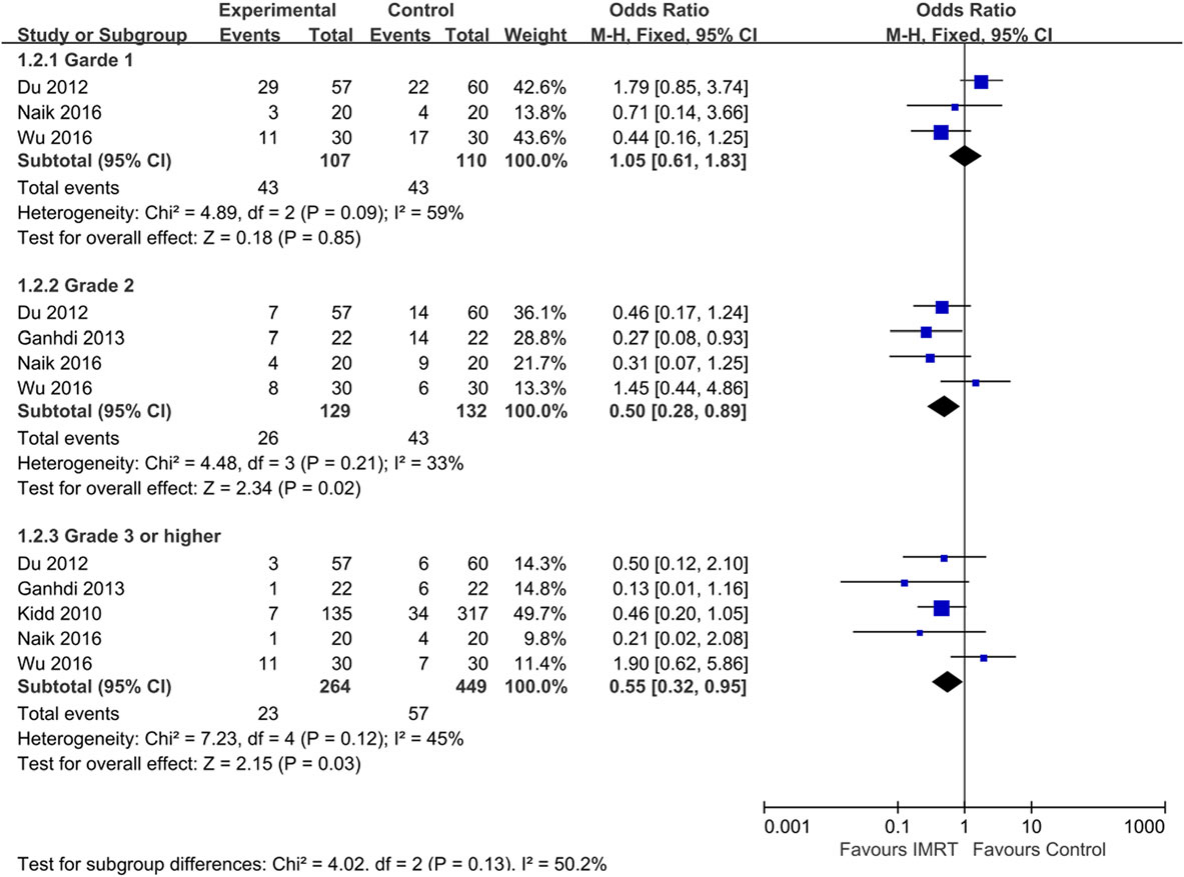
Comparison between IMRT and 2D-RT/3D-CRT for acute GI toxicity

Gastrointestinal (GI) and genitourinary (GU) were the most common side effects for cervical cancer patients treated with RT. A total of five studies reported instances of acute toxicity after patients received treatment, including acute GI and GU side effects. We analysed various grades of GI toxicity to assess the effect of treatment on patients. No statistical difference (OR = 1.05, 95% CI 0.61–1.83, *p* = 0.85) was evident, indicating that patients who received IMRT therapy exhibited more favourable outcomes than those who received 2D-RT or 3D-CRT therapy in terms of incidence of grade 1 acute GI toxicity. Additionally, the results suggested that patients in the IMRT group exhibited a lower incidence of grade 2 or higher acute GI toxicity than did those in the 2D-RT or 3D-CRT group (Grade 2: OR = 0.5, 95% CI: 0.28–0.89, *p* = 0.02; Grade 3 or higher: OR = 0.55, 95% CI: 0.32–0.95, *p* = 0.03; Fig. 3).

**Fig. 4:**
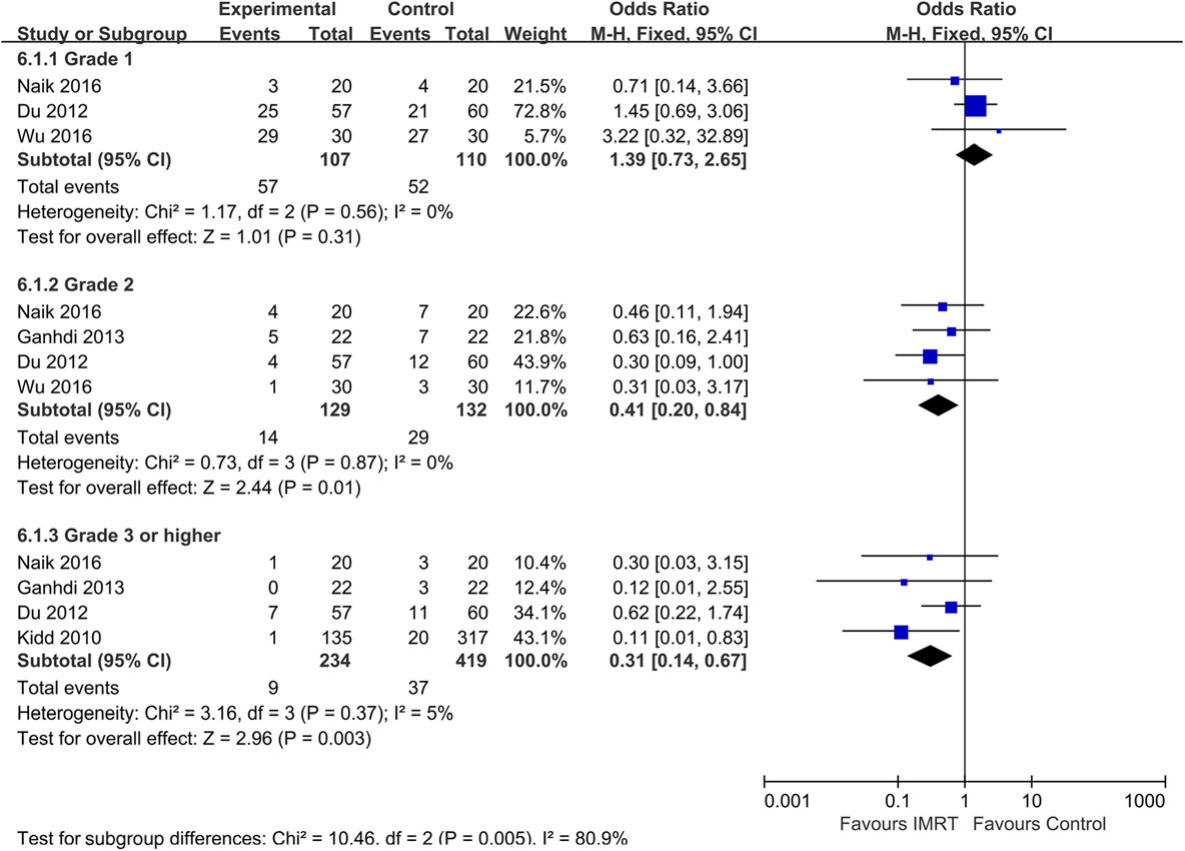
Comparison between IMRT and 2D-RT/3D-CRT for acute GU toxicity

Similarly, overall meta-analysis of the data revealed that the IMRT group was associated with a significantly lower incidence of acute grade 2 GU toxicity compared with the 2D-RT or 3D-CRT group (OR = 0.41, 95% CI: 0.2–0.84; *p* = 0.01). Pooled analysis revealed that incidence of grade 3 or higher GU toxicity among patients who received IMRT was significantly lower than that among patients who received 2D-RT or 3D-CRT (OR = 0.31; 95% CI: 0.14–0.67; *p* = 0.003; Fig. 4).

### Chronic toxicity

**Fig. 5:**
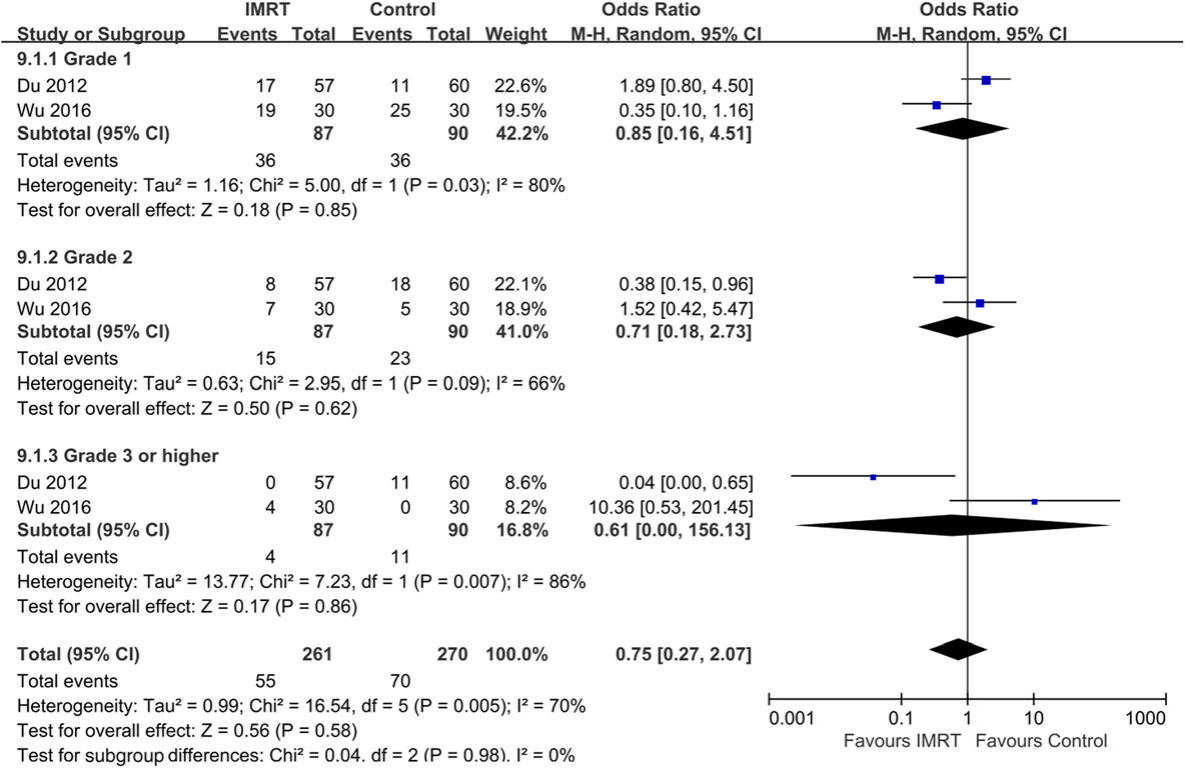
Comparison between IMRT and 2D-RT/3D-CRT for chronic GI toxicity

**Fig. 6:**
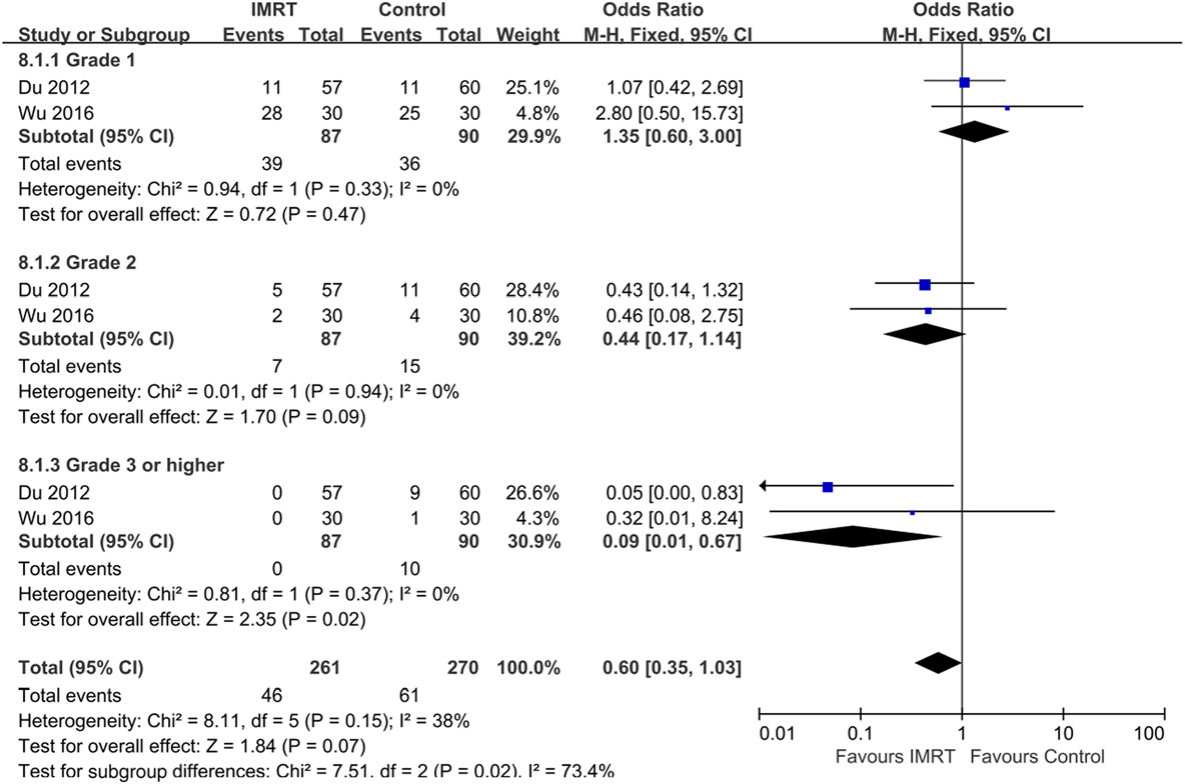
Comparison between IMRT and 2D-RT/3D-CRT for chronic GU toxicity

Two studies compared the chronic GI and GU toxicity exhibited by IMRT and control groups. According to our analysis, the trials were heterogeneous, and therefore a random model was chosen. No statistical significance was evident between the two groups in terms of chronic GI (Fig. 5). The incidences of grades 1 and 2 GU toxicity in the two groups were not significantly different (Grade 1: OR = 1.35, 95% CI: 0.6–3.0; *p* = 0.47; Grade 2: OR = 0.44, 95% CI: 0.17–1.14, *p* = 0.09). However, the incidence of grade 3 or higher GU toxicity in the IMRT group was significantly lower than that of the 2D-RT or 3D-CRT group (OR = 0.09, 95% CI: 0.01–0.67; *p* = 0.02; Fig. 6).

## Discussion

Pelvic RT combined with brachytherapy plays a critical role in the definitive treatment of patients with cervical cancer. With rapid developments in RT, IMRT has become widely used in treatment of cervical cancer, and it exhibits a dosimetric advantage because it can deliver a high dose of radiation to tumour tissue while restricting dose exposure of adjacent noncancerous tissues [15, 16]. However, because of the highly specific dose distribution in IMRT, the tumour target may be missed, especially in cases of cervical cancer.

The limitation of current imaging modality is that accurate tumour boundary demarcation cannot be ensured; thus, because of the anatomic specificity of the target location in cases of cervical cancer, organ motion may cause the target to be missed [17]. Therefore, the application of IMRT in cervical cancer treatment is highly controversial. Comparison of the curative effects of IMRT and conventional 2D-RT or 3D-CRT is crucial.

According to a 2012 systematic review and meta-analysis by Yang et al. based on 13 studies [18], IMRT significantly reduced the average proportion of irradiated volume of the rectum and small bowel compared with 3D-CRT in patients with gynaecologic malignancies. However, whether the dosimetric advantage of IMRT leads to more favourable clinical outcomes than those associated with conventional external beam radiation remains unclear. The pooled results of our meta-analysis indicated that IMRT application was associated with similar clinical outcomes to those of conventional RT (c-RT) in terms of both 3-year OS and 3-year DFS. However, Kidd et al. [13] reported a significantly greater OS for an IMRT group. This may be because the IMRT group had no lymph node involvement, which would have influenced survival rate. Only one relevant study [12] has reported 5-year progression-free survival rates (PFS) and 5-year OS. The results indicated a significantly higher 5-year PFS rate but no improvement in 5-year OS for an IMRT group compared with a c-RT control (5 year PFS: 64.9% vs. 44.3%, *p* = 0.03; 5-year OS: 71.20% vs. 60.30%, *p* = 0.064) for patients with advanced cervical cancer. These data are difficult to analyse and may not represent the true clinical outcomes for patients with cervical cancer. Thus, large-scale randomised trials are required to determine whether IMRT offers long-term survival benefits for women with cervical cancer.

RT exhibits curative effectiveness for cervical cancer in terms of tumour growth control, but the accompanying acute and chronic toxicities, which affect patient life quality, are of concern. Patients’ most common acute adverse reactions to RT are abdominal pain, varying degrees of diarrhoea, haemorrhage, intestinal obstruction, and granulocytopenia, and because of these potential side effects, some patients refuse RT [19]. Late toxicities may arise months to years after whole pelvis RT, and most commonly comprise intermittent diarrhoea; intolerance to certain foods; malabsorption of vitamins, lactose, and bile acids; and severe toxicities such as obstruction and fistulas [20]. Although the reported survival outcomes did not exhibit statistical difference between arms, we did observe a significant benefit with regard to toxicity. Our meta-analysis revealed that the frequency of acute grade 2–4 GI and GU toxicities and of chronic grade 3 GU toxicity was significantly lower in the IMRT group than it was in the control group. One study [10] did not provide the grades of toxicity and thus was not included in this portion of our analysis. A preliminary study indicated that IMRT was associated with less chronic GI toxicity than c-RT was in patients with gynaecologic malignancies [21]. However, this study involved limited follow-up and was based on patients with endometrial and cervical cancer, including those who had undergone surgery. In the present study, we determined that IMRT and c-RT exhibited no statistically significant difference in terms of chronic GI toxicities. However, in a study by Wu et al. [14]*,* a higher incidence of severe chronic GI toxicities was noted in patients who received IMRT compared with those who received 2D-RT, but the *p* value was not significant (IMRT vs. 2D-RT: Grade 3 = 13% vs. 0%, *p* = 0.054)*.* In addition, the number of studies indicating haematological toxicity is limited. In summary, these results indicate that IMRT offers considerable benefit in protecting at-risk organs and improving quality of life among patients with cervical cancer.

Our study involved several limitations. We included both prospective and retrospective studies, which introduced selection bias concerns. Additionally, only English-language publications were included, and thus language bias was probably introduced into the analysis. Moreover, most of the included studies were based on relatively small sample sizes. In addition, not all of the included studies compared clinical outcomes of IMRT groups with control groups, and most of them did not compare locoregional control rate (LRC) and PFS. Only one study provided 5-year PFS, which meant that this factor could not be evaluated in the present meta-analysis. Evidence in the literature was not conclusive enough to determine the efficacy of IMRT in the treatment of cervical cancer based on analysis of only OS, DFS, and toxicity. Additional high-quality clinical trials are warranted to verify the efficacy and benefits of IMRT for cervical cancer.

## Conclusion

To our knowledge, this was the first meta-analysis to compare the clinical outcomes and toxicity experienced by patients with cervical cancer who received definitive treatment with IMRT, 3D-CRT, or 2D-RT. This meta-analysis determined that IMRT was not superior to 3D-CRT or 2D-RT in terms of OS, but it was associated with relatively few instances of acute GU and GI toxicities. Regarding cancer control, further studies are required to determine the appropriate role of IMRT in cervical cancer management.

## Abbreviations

2D-RT: Two-dimensional radiotherapy
3D-CRT: Three-dimensional conformal radiotherapy
CI: Confidence interval
DFS: Disease-free survival
GI: Gastrointestinal
GU: gentiourinary
IMRT: Intensity-modulated radiotherapy
LRC: Locoregional control rate
ORs: Odds ratios
OS: Overall survival
PFS: Progression free survival
RT: Conventional radiotherapy
